# Opportunities and Barriers to Breast Cancer Screening in a Rural Community in Coastal Karnataka, India: A Qualitative Analysis

**DOI:** 10.31557/APJCP.2020.21.9.2569

**Published:** 2020-09

**Authors:** Thejas R Kathrikolly, Ranjitha S Shetty, Suma Nair

**Affiliations:** 1 *Department of Community Medicine, Kasturba Medical College, Manipal. Manipal Academy of Higher Education (MAHE), Manipal, Karnataka, India.*; 2 *Centre for Community Oncology, Kasturba Medical College, Manipal. Manipal Academy of Higher Education (MAHE), Manipal, Karnataka, India. *

**Keywords:** Breast cancer, barriers, focus group discussion, mortality, wellbeing

## Abstract

**Background:**

Breast cancer is reported to be the most common cancer among women in India with a high mortality to incidence ratio. Late presentation, driven by lack of awareness and limited accessibility to health services are some of the stated reasons for this. Given this context, this qualitative study was carried out to understand the perception of rural women towards the disease and factors that influenced utilization of available screening services among them.

**Methods:**

Forty-four rural women aged 20-60 years from a coastal province in southern India participated in four Focus Group Discussions (FGDs) that were conducted to understand their perception, attitudes and barriers towards breast cancer screening. Participants were identified from the community through purposive sampling and constituted of home makers and working women. The FGDs were led by trained facilitators and the discussions recorded. Ideas and concepts that emerged were listed as codes. Related and similar codes were grouped to form six themes.

**Results:**

Women in the study belonged to low- and middle-income households with a mean age of 42.8 **±** 7.8 years and almost all had attended school. Although the respondents exhibited fairly good knowledge about the disease, cultural inhibitions, forgetfulness, economic constraints and apprehension towards tertiary health care facility were some of the barriers reported in the uptake of screening services. Participants hailed the role of female health care providers as motivational figures and stressed the need for easily comprehensible information dissemination strategies besides expecting an equal participation of men in issues involving women’s health.

**Conclusion:**

Involving cancer survivors as educators and empowering men on women’s health in addition to the felt need of a patient advocate to improve accessibility were some of the highlights of the discussions. Addressing these could go a long way in improving the cancer care continuum in the region.

## Introduction

The rising burden of breast cancer in developing economies like India has been quite noticeable (Bray et al., 2018). This is further corroborated by the national cancer registries data that indicate not only a high age adjusted incidence rate but also a high mortality to incidence ratio (Malvia et al., 2017). This progressive increase in the incidence and mortality rates of the disease has been attributed to changing life styles owing to rapid urbanisation in a fast- growing economy (Chaturvedi et al., 2015). Awareness about the disease however, remains dismally low in the country especially among the vulnerable groups. This coupled with a non-existent organised screening programme results in delayed diagnosis and poor survival rates (Singh et al., 2015). Existing sporadic screening services on the other hand, are seldom used either due to prohibitive costs or prevailing cultural factors (Vidyarthi et al., 2013; Salian et al, 2015). Added to this is the inaccessibility of affordable treatment facilities and collectively these account for poor quality of life and survival rates among women with breast cancer in India. 

Early detection of breast cancer is crucial in improving survival rates (Anderson et al., 2003; Sathwara et al., 2017). While in developed countries three-fourth of the breast cancer cases get diagnosed at stage I or II, the converse is seen in most of the low- and middle-income countries (LMICs) (Ginsburg, 2013; Shulman et al., 2010; Rivera-Franco and Leon-Rodriguez, 2018). This disparity stems primarily from awareness deficit regarding the disease among women in LMICs besides other barriers such as stigma or fear towards cancers, discomfiture with screening procedures, gender bias and so on (Unger-Saldaña, 2014). This finding has been further reiterated through numerous studies conducted in India (Babu et al., 2013; Sathwara et al., 2017; Lakshmanan et al., 2017). In addition to the lack of an organised screening programme in the country, numerous socio-cultural barriers seem to play an important role in further dissuading women from getting timely screening or treatment (Singh et al., 2015; Yip, 2016). 

Understanding factors that aid utilisation of cancer screening activities could help design locally relevant and targeted awareness campaigns to bring about the necessary behaviour change. This qualitative study was undertaken with a view to determine the opportunities and barriers in the utilisation of existing screening services for breast cancer among rural women in southern India.

## Materials and Methods

*Participants and setting*

Women in the age group ranging from 20-60 years from Udupi taluk, a coastal province in the state of Karnataka in southern India were invited to participate in the Focus Group Discussions (FGDs). Participants were distributed across four villages in the region and belonged to low- and middle-income households and were literate. 

This being a coastal region, women are primarily occupied in small scale fish selling, fish curing, and other fish related activities, while other livelihood includes farming, small time business and beedi (tobacco) rolling. 

Unlike in the developed regions of the world there is no nation -wide organised cancer screening programme in India. Consequently, screening facilities like the mammogram are sporadic and offered mainly by private health care establishments that results in an out of pocket expenditure for the beneficiaries. It is also to be noted that these facilities are most often located at secondary or tertiary care centres that are not easily accessible to the rural population.

Philanthropic no cost screening initiatives like the one managed by the study team through a community oncology endeavour helps in creating awareness about the disease, which in turn facilitates women to access screening and diagnostic services. This endeavour includes a dedicated team that carries out awareness camps that are essentially planned community gatherings conducted with the intent of disseminating focused health information. Such camps are organised periodically by liaising with local government authorities, community organisations, leaders, volunteers and religious groups. Care is taken to reach out to the vulnerable and disadvantaged sections of the society. Over a period of time these activities have improved awareness regarding cancer in the region; however, late presentation of the disease continues to be of concern (Nair et al., 2016). 

*Research Team and Study Tool*

The team consisted of women researchers, two physician scientists (SN; RS) and a research scholar (TK) having more than five years’ experience in the design and conduct of qualitative studies. SN has published papers that used qualitative research methods. None of the researchers had any relationship with the study participants prior to conduct of the study. 

Professionally trained facilitators (women social scientists trained in FGD methodology) led the discussions ably supported by note-takers who made hand-written verbatim notes in English and the local language, Kannada in addition to audio recording the proceedings and constructing the sociogram. The investigators were present at the venue but restricted themselves to observing the proceedings. 

The FGD guide was developed in English by the research team based on existing literature and experts’ opinions. This was translated to the local language and pilot tested among 10 women participants. Inputs from the pilot study was used to make appropriate modifications and the facilitators used the modified guide to lead the participants through a discussion to understand their knowledge about breast cancer, its risk factors as well as attitudes and socio-cultural beliefs towards screening and best methods for educating rural women on the issue. Leading questions were avoided and appropriate probes were used wherever required to obtain relevant information.

*Focus Group Discussions*

Field health workers affiliated to the centre for community oncology encouraged women from the community to participate in the FGDs through face-to-face and telephonic contacts. Purposive selection was done to ensure that the group members were heterogeneous with respect to socio-demographic characteristics. Women who accepted the invitation were informed about the date, time and venue of the FGD to be held in their area. Of the fifty women who were approached, six dropped out citing time constraints, household engagements, hesitancy, shyness and lack of interest as reasons.

The FGDs were conducted at rural health centres situated in the respective village and ten to twelve participants were present during each session of the FGD. Non participants were not entertained and accompanying persons if any were requested to remain in the waiting area of the health centre till the completion of the session. One FGD was conducted in each village amounting to a total of four FGDs comprising of forty-four participants in all. The total number of FGDs were restricted to four in view of data saturation. 

Each FGD started with a round of introduction and an ice-breaking activity to warm up the discussions. Following this the facilitators introduced the topic and outlined the objectives prior to initiating the discussion. Participants were encouraged to freely express their views as this being a discussion there were no right or wrong answers and the information provided was confidential. A pre-designed questionnaire was administered prior to the FGD to assess their general demographic characteristics. The FGD sessions lasted between 90-120 minutes and ended at a point where further knowledge was not elicited. At the end of each FGD, findings were summarised and participants briefed. 

*Data analysis*

Discussions were audio recorded and extensive field notes were prepared. These were translated and transcribed verbatim. Manual thematic analysis was carried out to analyse the study findings. The transcripts were read independently by two reviewers multiple times in order to capture and group similar emerging ideas. Ideas and concepts that emerged were listed as codes. Both the authors compared the code list and any discrepancy between the authors were resolved by discussion until consensus. Related and similar codes were grouped into code families to form sub-themes and themes. Vernacular words were retained, thus avoiding dilution of their essence in the respective contexts during the discussions. Themes consisted of detailed descriptions of participants’ belief and practices related to breast cancer and the factors that influenced uptake of screening services. Authors then provided interpretations to these themes.

*Ethical Considerations*

The study protocol was approved by the institutional review board (Registration no: ECR/146/Inst/KA/2013; Project Approval no.: IEC 172/2015) prior to initiation of the study. Informed written consent was obtained from the participants that included consent to audio record the proceedings and publish the data anonymously. Care was also taken to ensure participant anonymity throughout the study process by assigning them serial numbers and confidential handling of data. 

## Results

Women in the study belonged to low and middle-income households. All were literate with almost three quarter of the participants having attended secondary level of schooling or higher, which illustrates the high literacy status of the region. Demographic and reproductive characteristics of the study participants are as shown in Table 1. Nearly two third of the women were homemakers with a mean age of 42.8 + 7.8 years. Mean age at menarche and first live birth was 14 + 1.6 years and 24 + 3.3 years respectively. 

Six themes emerged from this study following the qualitative data analyses, as illustrated in Table 2. What follows is a summary of these themes:

*i. Health concerns of women*

Women reported conditions such as vaginal discharge, knee pain, breast cancer, prolapsed uterus, fibroids and various aches and pains as their main health concerns. Although they acknowledged the presence of rural health care centres in their respective areas, some of the women admitted to not utilising the available services specially for conditions related to the breast and the reproductive tract due to hesitancy and shyness in talking about these diseases and being scared about the consequences on finding an abnormality.

Self -examination of the breast, regular clinical examination, maintaining hygiene, in addition to a prudent diet and adequate physical activity emerged as important self-care activities to keep these diseases at bay. “*We should get screened regularly so that the condition can be treated at an early stage, otherwise it is difficult to treat*” was their opinion regarding early detection. 

Women bore a considerable amount of fear while discussing the term cancer. One of them coined the phrase: *“cancer means cancel” *as an accepted mindset among most rural people. Cancer was perceived to be a major disease, requiring expensive treatment resulting in poverty and early death. For the same reasons, they were also apprehensive of being referred to a tertiary care hospital for further treatment. 

Women exhibited reasonable knowledge regarding breast cancer and its early detection. Excerpts of participant responses regarding this are summarized in Table 3. A lump in a breast can mean cancer and this can be prevented by regular screening was the general understanding. 

*ii. Barriers to breast cancer screening*

Participants agreed that screening for breast cancer helps in its early detection and thus can help in better treatment. However, it was stated that a woman may just ignore the disease till the last stage simply due to the fear of the impending financial pressure that the treatment may bring upon her family: *“…what can be done if detected early, it’s a long process and have to spend a lot of money after that for treatment. So, no use of early detection!”*

The underlying stigma about breast cancer was primarily about hesitation and inhibition in consulting a doctor especially given the circumstance of young age of these women (Table 3). Some of the participants believed that breast cancer signified “*Naga threat*” (wrath of the snake god) and suggested home remedies like “*Kashaya*” (locally made herbal brew), “*herbal oil*” and “*green leaves*” for treatment of the disease. A few stated that since it was perceived to be hereditary, a family member having the disease could affect the marriage prospects of female members adversely. “*Being poor itself is a stigma*” was an important remark that was associated with the low economic background of some women.

“*Forgetfulness*” and “*lack of time*” emerged as major constraints in adopting screening procedures like breast self-examination (BSE). Some women mentioned the issue of privacy especially in a joint family and small household space as a hindering factor to practice BSE, while others mentioned that they remember only when they hear about a cancer case. 

When queried about screening by mammogram, most of the women mentioned accessibility as a concern as the tertiary care centre that provides the facility is far from their area of residence: “*Reaching the centre is difficult, very confusing and it would be helpful to have a contact person at the centre, who could be reached on a mobile phone*”. Participants believed the tertiary care centre to be rather large and easy to get lost without assistance. Some others suggested that if transportation facilities were provided by the health care provider, it could further enhance accessibility. Another concern was the presence of male health care providers as that they felt would inhibit them. Almost all the participants stated that they felt more comfortable discussing these issues with a lady health care provider. 

*iii. Factors motivating the uptake of screening facilities*

Participants pointed out that cancer survivors were the right people to motivate others to talk about the disease. There was also a felt need for women to be made aware about the seriousness of the disease and its effect on the whole family if neglected. On being asked how women could be motivated to be more proactive, they opined that awareness camps and FGDs similar to the one they were currently attending could go a long way in achieving these. Women also expressed that “*financial help*” through charity organisations would further encourage women to seek help at the right time.

A good number of participants declared doctors, especially lady doctors to be the most suitable to spread awareness about breast cancer. Nurses came a close second to instil confidence and put people at ease. Respondents also endorsed the encouraging role that grass root level health workers like the Anganwadi workers (AWWs) and Accredited Social Health Activists (ASHAs) played in bringing about positive health behaviour among people. Almost all the participants appeared to be wary of trainee health care providers like medical or nursing residents being entrusted with this very important task as they presumed, these personnel lacked the experience and the rapport that was so essential to influence group behaviour change. 

*iv. Perception of cancer prevention and control initiatives*

The recent initiative by the government of India to promote opportunistic screening of non- communicable diseases including common cancers is a welcome measure. However, its uptake and implementation has been patchy. Moreover, there is limited financial outlay and facilities for diagnostics and treatment within the programme. All the same there are certain health schemes promoted by the government for the socially disadvantaged groups. For the most part, participants lamented about the lack of information regarding government led cancer control initiatives. Some of the participants, who were serving as AWWs mentioned the occasional cancer camps that were held at the district hospital for the benefit of the underprivileged.

*v. Information dissemination*

Women emphasized the importance of using pictorial messages to disseminate information. Participants stated that the best way was to use pamphlets and videos in an entertaining fashion. It was felt that highlighting pictorially the differences between the normal and the diseased state would enable women to detect abnormalities themselves early on. 

Participants believed that educational materials should contain myth busters and make them understand the risk factors for the disease. Moreover, women considered it to be useful if the messages outlined the action plan that one was expected to adopt if diagnosed with cancer. It was also opined that the messages should not be frightening, as scary pictures could act as a deterrent. One of the participants expressed, “*(educational material) should not scare people, should give hope and not tell that there is no cure*”. 

Nearly all groups backed cancer survivors to spread awareness messages as they felt that it would be most encouraging. As regards the chosen mode for conveying such messages, the collective mandate was for incorporating these messages during popular television serials as television viewing was a widespread pastime among the women folk of the region. The idea of including it in the daily newspaper did not have too many takers as not many of the women were in the habit of reading the newspaper. Other proposed modes were health talks, interactive street plays and the radio, although many felt that more people watched television rather than listening to the radio.

*vi. Role of men in women’s health*

Unlike in most parts of the country some communities in this region practice a matriarchal system. Despite this, men play a significant role in deciding when and where women should access health care. While the participants strongly backed the notion that their men folk should be educated about women’s health, they suspected that the attendance would be dismal and therefore, the exercise futile. Notwithstanding this, women were certain that those who attended could spread the message and bring about a small change: *“Husbands and fathers taking a more active role could bring about a positive change, as in most instances they were the primary decision makers in the family*”. Notably, participants argued that sensitising men to women’s health issues would completely rule out any necessity for women to be hiding such facts from them, thereby ensuring timely care and assistance to prevent and cure the disease. Some of these responses are summarised in Table 4.

**Table 1:** Baseline Characteristics of Participants (n=44)

Variables	Number (%)
Educational qualification	
< 5 years of schooling	02 (4.5)
5-12 years of schooling	35 (79.6)
Graduate	07 (15.9)
Occupation	
Housewife	29 (65.9)
*ASHA / AWW worker	10 (22.7)
Others	05 (11.4)
Age at marriage (in years)	
16-19	11 (25.0)
20-24	23 (52.3)
>24	10 (22.7)
Aware of Breast Self- Examination	36 (81.8)
Practicing Breast Self -Examination	33 (75.0)
Aware of mammography	32 (72.7)
Undergone mammography	17 (38.6)

*ASHA, Accredited Social Health Activist; AWW, Anganwadi Worker

**Table 2:** List of Themes and Codes that Emerged from the Study

Themes	Codes
Health concerns of women	i. Breast and urogenital related concerns ii. Utilisation of health services iii. Fear iv. Regular screening v. Hesitancy and shyness
Barriers to breast cancer screening	i. Myths ii. Stigma iii. Financial pressure iv. Hesitation v. Poverty vi. Lack of time/ privacy vii. Accessibility to treatment facilities viii. Presence of male health workers ix. forgetfulness
Factors motivating the uptake of screening facilities	i. Motivating figures ii. Awareness camps iii. Philanthropic support i. Government led cancer control initiatives ii. Lack of information iii. Cancer camps for the under- privileged
Perception of cancer prevention and control initiatives	
Information dissemination	i. Mass media ii. Cancer survivors iii. Health talks iv. Interactive street plays
Role of men in women’s health	i. Educating men ii. Disinterested men folk iii. Sensitising men to women’s health issues

**Table 3:** Participant Perspectives and Perceived Barriers

FGD 1: P3	*“There is good facility in the area but ladies are not utilizing the services. This is because women are hesitating to talk about breast cancer and also, they are scared of finding some abnormality”*
FGD2: P11	* “chances of survival are minimal”*
P12	* “If there is a lump in the breast it can be cancer, it can be detected by checking regularly”*
FGD 3: P11	*“Big disease, no cure, Expensive treatment, might die early, early detection could help, hair would fall”*
FGD 1: P10	*“Being poor itself is a stigma”*
FGD 2: P8	*“Women feel shy and hesitate to go to doctors”*
FGD 4: P6	*“…we feel lost when we reach the big hospital. If there is a known person at the big hospital, it is more convenient”*
P12	*“Scared of finding that something is wrong”*
FGD 4: P8	*“Feel there is no cure for cancer”*
FGD 1: P8	* “It would be good if women who have already undergone the test can motivate other women”*

FGD, Focus Group Discussion; P, Participant

**Table 4:** Participants’ Beliefs on Male Engagement in Women’s Health

FGD 1: P4	*“Try to talk to males regarding women’s health so that they understand. Can organize camps to create awareness among males also”*
FGD 2: P2	*“Should involve men in health education” *
P11	*“Men do not involve – they feel it is a waste of time”*
FGD 3: P8	*“Some men do not give importance to women’s health”*
P10	*“They can be given the same information that we receive”*
FGD 5: P5	*“Men should be educated about breast cancer, so that they encourage women to go for the test”*

FGD, Focus Group Discussion, P, Participant

## Discussion

Gupta et al., (2015) in their review on breast cancer awareness among Indian women reported the dearth of qualitative studies pertaining to this issue in the Indian context. Given this backdrop the present data gains significance. 

The group of participants had considerable knowledge about the importance of early detection in breast cancer, including the various early detection procedures in spite of their rural low economic background. However, this did not encourage them to seek medical help when needed, a factor driven by cultural values and social stigma. A study that explored the possible barriers to breast cancer screening in two metropolitan Indian cities reported that in spite of an easy access to healthcare facilities and a good educational background, considerable number of participants cited embarrassment as one of the reasons to not access screening facilities (Vidyarthi et al., 2013). This prompts us to understand that seeking screening services or medical help is strongly conditioned by cultural values and beliefs of the population. In another qualitative study among Asian Indian women (Wu et al, 2012), ‘*fear*’ and aversion towards the word ‘*cancer*’ were predominant elements regarding perception of the disease. These findings are in agreement with the sentiments expressed by the participants of our study. Given the rising incidence of breast cancer in India, the need for an organized screening program cannot be overstated (Singh et al., 2015). The same was also expressed by the participants of this FGD.

Literature has indicated stigma to be a major barrier to breast cancer screening. Studies (Singh et al., 2015; Nyblade et al., 2017) have shown that the fear of being disregarded by the community or spouse, inhibited women from seeking medical help following the detection of a lump. These findings are in concordance with that of the women in this study, who affirmed that marriage prospects for them and their family members would be at stake if they were detected with a lump.

Some studies have reported ‘*cost*’ ‘*shyness*’ and ‘*fear*’ among other factors as barriers to screening (Lyttle and Stadelman, 2006; Dey et al., 2016). These were echoed by our participants as well, who expressed fear of impending costs of treatment, embarrassment and social stigma as factors that prevented them from accessing screening services. The study by Dey et al., (2016) reported that being diagnosed with a breast anomaly amounted to the woman being branded as immoral, which in turn prevented her from seeking help. This fortunately did not seem to be a concern among women in this region and this probably is a reflection of the relatively higher education and living standard that this southern Indian province boasts of (“*Udupi District Human Development Report - 2014,*” 2014).

Our study also supports the finding that South Asian women though reasonably informed are seldom proactive about their health in comparison to their Western counterparts (Scanlon and Wood, 2005; Forbes et al., 2011; Vidyarthi et al., 2013). The aspect of ‘*lack of time*’ as a barrier has also been evidenced by Dey (2016) (Dey et al., 2016). This is adequately illustrated in our participants’ claim of ‘*family pressure*’ and ‘*forgetfulness*’ due to household chores as reasons for not carrying out preventive breast health measures. ‘*Forgetfulness*’ especially, was one of the important factors previously identified in the same community setting as one of the barriers to self- breast examination (Rao et al., 2005). Given the rural and low socio-economic background of the participating women, they felt the need to be made aware of a guided referral method by which they could easily access the facility of mammogram. 

It has been shown that involving trained healthcare workers in screening activities increases community participation (Singh et al., 2015). This has been further reaffirmed by Suwankhong and Liamputtong (2018) on Thai migrant women and Lyttle (2006) on Appalachian women. On a similar note, participants of this study suggested that the involvement of a ‘lady doctor’ during screening programmes acted as a motivational booster. Wu et al., (2012) in their study showed that involvement of family and friends had a positive bearing on women’s outlook about the disease. This finding bears semblance to ours wherein the women felt that active role of family members in their health issues would motivate them to participate in screening activities.

The participants recommended pictorial messages as an enabler to understanding the disease process. Women in this study were partial to the television medium, which is in contrast to the findings by Lyttle and Stadelman (2006), where women had no particular preference and appeared to be equally at ease with all forms of mass communication. The TV preference indicates that this could be an effective medium to disseminate information in this region.

Studies from Mumbai and Delhi have observed that instilling awareness among men about breast cancer would motivate them to play a proactive role in helping women to seek timely medical help (Salian et al., 2015; Dey et al., 2016). This was echoed by the women in our study. 

It must be noted that the women in this study have a higher literacy rate in comparison to a number of regions across India. The observations recorded here may therefore, vastly differ from other parts of the country. Additionally, it is also possible that during certain discussions dominant personalities may have had an effect on introverts thus dissuading expression of true beliefs and ideas.

Participants of this study were enthusiastic to know more and suggested pragmatic methods to improve the uptake of screening among this population. There was a felt need to have a liaising person akin to a patient advocate between the community and the tertiary care hospital as rural women most often feel intimidated by large hospital settings and avoid visiting the place all together. There was also a collective call for involving men proactively as that they felt would go a long way in ensuring better health seeking behaviour among women.
